# Functional Connectivity of the Anterior and Posterior Hippocampus: Differential Effects of Glucose in Younger and Older Adults

**DOI:** 10.3389/fnagi.2020.00008

**Published:** 2020-01-31

**Authors:** Riccarda Peters, David J. White, Brian R. Cornwell, Andrew Scholey

**Affiliations:** ^1^Centre for Human Psychopharmacology, Swinburne University of Technology, Melbourne, VIC, Australia; ^2^Centre for Mental Health, Swinburne University of Technology, Melbourne, VIC, Australia

**Keywords:** resting-state fMRI, glucose, spatial navigation, hippocampus, aging, cognition

## Abstract

The hippocampus features structurally and functionally distinct anterior and posterior segments. Relatively few studies have examined how these change during aging or in response to pharmacological interventions. Alterations in hippocampal connectivity and changes in glucose regulation have each been associated with cognitive decline in aging. A distinct line of research suggests that administration of glucose can lead to a transient improvement in hippocampus-dependent memory. Here, we probe age, glucose and human cognition with a special emphasis on resting-state functional connectivity (rsFC) of the hippocampus along its longitudinal axis to the rest of the brain. Using a randomized, placebo-controlled, double-blind, crossover design 32 healthy adults (16 young and 16 older) ingested a drink containing 25 g glucose or placebo across two counter balanced sessions. They then underwent resting-state functional magnetic resonance imaging (rs-fMRI) and cognitive testing. There was a clear dissociation in the effects of glucose by age. Magnitude change in rsFC from posterior hippocampus (pHPC) to medial frontal cortex (mPFC) was correlated with individual glucose regulation and gains in performance on a spatial navigation task. Our results demonstrate that glucose administration can attenuate cognitive performance deficits in older adults with impaired glucose regulation and suggest that increases in pHPC-mPFC rsFC are beneficial for navigation task performance in older participants.

## Introduction

Increasing the levels of available glucose, by the administration of a glucose drink, has been shown to improve cognitive performance in both younger and older adults in the minutes and hours following the drink (Smith et al., 2011). The effects of glucose have been reported to be comparable to those observed after administration of pharmaceutical cognitive enhancers (Riby, 2004).

Converging evidence suggests a relationship between the effect of glucose on tasks which are predominantly related to hippocampal function such as episodic memory and spatial memory (Riby, 2004; Smith et al., 2011).

The hippocampus is known to be important in the formation and recollection of memory (Moscovitch et al., 2005) and is a key brain hub for episodic memory, operating in the context of a large-scale network (Nyberg et al., 2000). Resting-state functional connectivity (rsFC) analysis, including using functional magnetic resonance imaging (fMRI), has proved to be a powerful tool to help unravel the functional architecture of brain networks (Raichle et al., 2001). One of the most common findings in studies of age-related rsFC is an association between advancing age and decreased functional connectivity within the default mode network (Ferreira and Busatto, 2013) and overall reductions in functional connectivity between the hippocampus and the rest of the brain (Geerligs et al., 2015).

It is increasingly recognized that there is a structural and functional dissociation between anterior and posterior segments of the hippocampus (Strange et al., 1999). This includes distinct patterns of rsFC displayed by anterior (aHPC) and posterior parts (pHPC) of the hippocampus (Wagner et al., 2016). The functional relevance of these networks and age-related changes therein are largely unknown. There is mixed evidence regarding the association between pHPC and aHPC connectivity and performance on cognitive tasks, and the functional differentiation of aHPC and pHPC is yet to be clearly defined. An episodic-spatial dichotomy of anterior and posterior hippocampal segments has been proposed, with pHPC being related to spatial memory functions and aHPC to episodic memory functions (Persson et al., 2018).

Much of the work on the influence of glucose on neurocognitive performance has focused on age-related effects (van der Zwaluw et al., 2015). Senescence is accompanied by changes in glucose metabolism (Blesa et al., 1997), specifically poorer glucose regulation. These changes in glucose regulation have been linked to age-related cognitive decline (Korol and Gold, 1998; Awad et al., 2004) and Alzheimer’s disease (Watson and Craft, 2003).

While cognitive domains implicated in the glucose facilitation effect have been argued to preferentially enhance hippocampus-dependent tasks, relatively limited research has directly explored underlying neurophysiological mechanisms in the human brain. Several event-related potential (ERP) studies support the involvement of the hippocampus (Smith et al., 2009; Brown and Riby, 2013; Scholey et al., 2015). Further evidence for the involvement of the hippocampus in the effect stems from a study using fMRI (Parent et al., 2011).

To the best of our knowledge, no study to date has considered the anterior-posterior division of the hippocampus in the study of the glucose facilitation effect. Furthermore, rsfMRI has not been directed to compare the modulation of cognition and resting-state connectivity in younger and older adults. The present study addresses these gaps in the literature. Here, we describe the outcomes of a placebo-controlled, double-blind, crossover neuroimaging study investigating the relationship between age, glucose and human cognition with a special emphasis on the connectivity of the hippocampus along its anterior-posterior axis to the rest of the brain.

## Materials and Methods

### Participants

A total of 32 healthy right-handed participants from Melbourne, Australia, were recruited for this randomized, double-blind, crossover trial. Half of this group (*n* = 16, eight women) consisted of younger subjects (mean ± SD: age 25.8 ± 3.2 years, range 21–30) and the other half (*n* = 16, eight women) consisted of older subjects (mean ± SD: age 68.6 ± 6.54, range 55–78). The participants were recruited *via* flyers, online advertising and from a database. All participants provided informed consent and received a small monetary compensation for their participation. The study was approved by the Swinburne University Ethics committee and all procedures were performed in accordance with the principles of the 1974 Declaration of Helsinki.

Inclusion criteria included normal or corrected-to-normal vision and hearing, no major physical illness and had no history of neurological/psychiatric illness or head trauma. Further exclusion criteria were a diagnosis of diabetes mellitus, a history of hypersensitivity to glucose, heart disease or high blood pressure, smoking, substance abuse, intolerance to artificial sweeteners, pregnancy, claustrophobia, metal implants or any other contraindications to MRI.

Participants were excluded if they reported health conditions that would affect food metabolism including the following: food allergies, kidney disease, liver disease and/or gastrointestinal diseases (e.g., irritable bowel syndrome, coeliac disease, peptic ulcers).

Subjects were also excluded if they were taking any medication, herbal extracts, vitamin supplements or illicit drugs which might reasonably be expected to interfere with blood glucose levels within 4 weeks prior to and during the study. In order to study associations with glucoregulation, the study included a range of fasting blood glucose levels (mmol/l). Fasting levels above 6 mmol/l were presumed to reflect fasting compliance with compromised glucoregulation as long as they were noted consistently across visits. Where fasting levels above six were not noted across both visits, the subject was excluded from the analysis as this was taken as evidence of non-compliance with fasting.

During the testing session, participants could be excluded if they scored lower than 25 on the Mini-Mental State Exam (MMSE; Folstein et al., 1975). Participant enrolment and inclusion pathways are depicted in a CONSORT flow diagram in the Supplementary Figure S1.1. All testing occurred at the Centre for Human Psychopharmacology at Swinburne University.

### Procedure

Participants attended the Centre for Human Psychopharmacology on three occasions a screening visit and two experimental sessions (with the experimental sessions balanced for treatment order). At the screening visit, informed consent was obtained, and eligibility was confirmed. Socio-demographic and morphometric data were collected (see Table 1). The session also served to familiarize participants with the cognitive tasks they would encounter during the study days.

**Table 1:** Demographics.

	Young	Older	*P*-value
*N* (female)	16 (8)	16 (8)	-
Age (years)	25.0 ± 3.5	68.3 ± 6.4	-
BMI (m/kg^2^)	23.0 ± 4.5	25.0 ± 4.01	0.224
Education (years)	17.0 ± 2.0	15.4 ± 3.6	0.117
MMSE	29.8 ± 0.3	29.2 ± 1.0	0.025*

Note: for BMI, education and MMSE numbers represent: mean (standard deviation), significant results are marked *at 0.05 level.

The two experimental sessions started between 8.30 am and 10 am and were scheduled between 2 and 14 days, apart. Participants were asked to abstain from food and drink (except water) after 10 pm before testing (to achieve a 12-h overnight fast). Participants were questioned on compliance and excluded in case of non-compliance. Fasting blood glucose levels were measured *via* capillary fingerprick using a Freestyle Optium Blood Glucose Sensor and Optium Blood Glucose Test Strips (Abbott Diabetes Care Limited, Witney, UK) according to the manufacturer’s instructions. Following baseline glucose measurement, participants were asked to complete self-report questionnaires measuring mood and appetite. The self-report questionnaires were administered after each blood glucose measurement throughout the testing day and will be described elsewhere. They then received the treatment drink which consisted of 25 g glucose (Glucodin Pure Glucose Powder) mixed with 150 ml of water and 20 ml of sugar-free cordial in the glucose condition, and two tablets (30 mg) sodium saccharine (Hermesetas^©^) mixed with 150 mL of water and 20 ml sugar-free cordial in the placebo condition. It has previously been shown that the two drinks are indistinguishable in taste and mouthfeel (Scholey et al., 2001; Scholey and Fowles, 2002). There is evidence that the glucose facilitation effect follows an inverted U-shape dose-response curve in humans (Parsons and Gold, 1992), and 25 g has been reported as the optimal dose for memory enhancement (Riby, 2004).

Participants were randomly assigned to a treatment sequence that counterbalanced the order of treatments within age groups and gender. To ensure blinding, randomization and preparation of the drink were performed by a disinterested third party with no other involvement in the study. Blood glucose was measured again 20 min, 120 min, and 150 min post-ingestion by a person who was not the experimenter to maintain blinding (unblinding occurred only after data analyses were completed).

**Figure 1:**
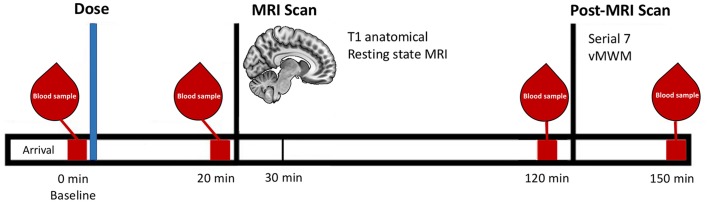
Schematic representation of the testing timeline for the treatment visits.

MRI scanning commenced 30 min post-ingestion. This interval was selected to ensure that blood glucose levels would be maximally elevated during MRI scanning (Korol and Gold, 1998). Anatomical (T1) images were acquired first, followed by a seven-minute resting-state scan (Figure 1).

### Cognitive Testing

Cognitive testing took place after MRI scanning (120 min post-ingestion; Figure 1). Two cognitive tests focusing on different domains of memory were used, as follows.

#### Working Memory

Working memory performance was assessed using the mental arithmetic Serial Sevens task. It involves the serial subtraction of seven from a given number. Previous work has shown that performance on this to be enhanced by glucose administration (Scholey et al., 2001). Standard instructions were displayed on a computer monitor, informing the participant to count backward in sevens from the given starting number, as quickly and accurately as possible, using the numeric keypad to enter each response. Performance was assessed using the number of correct subtractions within 2 min.

#### Spatial Learning and Memory

Spatial learning performance was measured using a virtual analog of the Morris Water Maze (vMWM; Morris, 1981). The latter has been used extensively in rodents to study hippocampal-dependent spatial navigation. The vMWM has been validated for use in humans (Cornwell et al., 2008).

**Figure 2:**
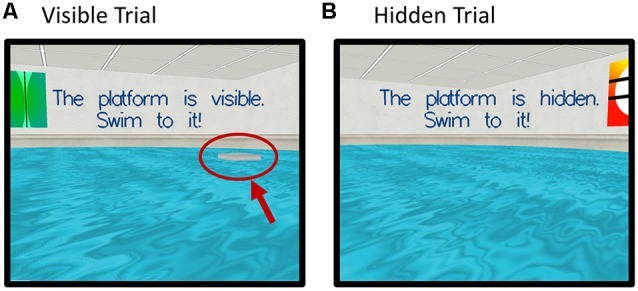
Screenshots of the virtual Morris Water Maze Task (vMWM) which was presented to the participants on a computer screen. Panel **(A)** depicts a trial where the platform is visible (red arrow and circle were added retrospectively and are not part of the actual display). Panel **(B)** depicts a trial where the platform is hidden (“submerged”).

Participants were instructed to try to find a platform in a virtual pool environment presented on a computer screen. The platform was visible on some trials and hidden (“submerged”) on others (Figure 2).

Probe trials were administered in which the platform was removed unbeknownst to participants. In this case, participants started from a novel position in the pool in order to assess spatial memory performance beyond immediate training experience. The platform was then moved to a new location and participants completed an additional set of hidden platform trials along with a second probe trial. The dependent measure was heading error (square-root transformed) or angular deviation from a straight path to the platform’s location on the probe trials.

For detailed task descriptions see section 2 in Supplementary Material.

### MRI Acquisition

Participants underwent MRI scanning on both testing days, following the same protocol. fMRI data were acquired using a 3-Tesla Siemens Magnetom Trio scanner (Siemens, Erlangen, Germany) at Swinburne University of Technology, Melbourne, Australia with a 32-channel head coil. To minimize head movement comfortable padding was placed around the participants’ head and participants were instructed to lie as still as possible. Structural high-resolution 3D T1-weighted magnetization prepared rapid acquisition gradient echo (MP-RAGE) anatomical images for anatomical reference were collected at the start of the scanning session (1 mm isotropic MP-RAGE, TR = 1,900 ms, TE = 2.52 ms, flip angle = 9°). Following the anatomical image, the participant underwent 7 min 15 sec resting-state fMRI (rsfMRI), during which they were instructed to keep their eyes open and look at a fixation cross. Functional data were obtained continuously with an interleaved multi band sequence (multiband acceleration factor = 6, bandwidth = 1,860 Hz/Px, TR = 870 ms, TE = 30 ms, echo spacing = 0.69 ms, flip angle = 55°, field of view = 192 mm, voxel resolution = 2 × 2 × 2 mm, slice orientation = transversal, number of slices = 66,500 volumes per session). MRI scanning took 60 min in total and also included two task-related fMRI scans and a spectroscopy sequence that will be reported elsewhere.

### Resting-State Analysis

Functional and structural images were processed using the CONN toolbox Version 17f (Whitfield-Gabrieli and Nieto-Castanon, 2012,^1^) for Statistical Parametric Mapping Software (SPM12; Wellcome Department of Imaging Science, Functional Imaging Laboratory, University College London) run under Matlab R2014a. Preprocessing steps were conducted using the default preprocessing pipeline for volume-based analysis (to MNI space; realignment and unwarping, ART-based identification of outlier scans for scrubbing, simultaneous gray matter (GM), white matter (WM), and cerebrospinal fluid (CSF) segmentation and normalization into standard MNI space (Montreal Neurological Institute, Montréal, QC, Canada).

To remove confounding effects from the BOLD time series the anatomical CompCor strategy (Behzadi et al., 2007) was used as implemented in the CONN toolbox. Physiological and other spurious sources of noise were estimated and regressed out of the BOLD functional data in the denoising step (simultaneous option). Five principal components were extracted from both WM and CSF, as well as 12 motion regressors (six head motion parameters + six first-order temporal derivatives) derived from spatial motion correction, which were used as temporal covariates and removed from the BOLD functional data using linear regression.

The resulting residual BOLD time series were band-pass filtered with a frequency window of 0.008 Hz–0.2 Hz.

In addition to the above steps controlling for motion, an analysis of motion across groups and conditions was conducted. Framewise displacement (FD; maximum total and averages scan-to-scan) was calculated according to Power et al. (2012) between-sessions and between-groups (see section 3.1 in Supplementary Material). As FD was higher for younger adults than older adults at both treatment sessions, and FD also differed between sessions in the younger group, all group-level analyses included average framewise displacement as a covariate at the second level.

### Seed Based Connectivity Analysis

To assess rsFC of the hippocampus, four seed regions were defined based on coordinates which have been used in previous research (Wagner et al., 2016) to facilitate comparisons of results. Four ROIs were created in MarsBar toolbox^2^ and defined as spheres with a 5 mm radius around the anterior and posterior part of the hippocampus on the left (anterior: *x* = −28, *y* = −12, *z* = −20; posterior: *x* = −28, *y* = −24, *z* = −12) and right side (anterior: *x* = 28, *y* = −12, *z* = −20; posterior: *x* = 32, *y* = −24, *z* = −12). The resting-state BOLD signal time-series of each hippocampal region of interest was extracted and correlated against voxels of the rest of the brain for each session of each subject. Fisher z transformation was applied.

### Statistical Analysis

Blood glucose level and cognitive outcomes were assessed by mixed-design ANOVA, with treatment as a repeated-measures factor and age as a between-subjects factor (IBM SPSS Statistics, Version 24). Blood glucose levels had additional repeated measures factor of assessment time within sessions. Glucose tolerance was assessed as an incremental area under the curve (iAUC) using the trapezoidal rule (Le Floch et al., 1990) based on the four blood glucose measurements taken at baseline, 20 min, 120 min and 150 min post glucose administration. Higher iAUC values reflect higher circulating glucose levels, indicative of poorer glucoregulatory ability.

Functional connectivity analyses used whole-brain voxel-wise mixed within-subject (glucose, placebo) and between-subject (younger, older) second-level models, using the partitioned variance approach implemented in CONN, in order to test for treatment × age-group interactions. All rsFC analyses used a cluster-extent FWE-corrected *p*-value < 0.05, obtained using non-parametric statistics with 5,000 permutations at a cluster-defining threshold of *p* < 0.001.

To explore the relationship between changes in rsFC and performance on cognitive measures, *post hoc* Pearson’s correlation coefficient analyses were carried out using SPSS.

## Results

Data from two participants of the young group (one male, one female) was omitted from the analysis because they showed significantly higher fasting glucose levels at a single session compared to the other session. The higher fasting glucose levels could indicate non-compliance to the fasting regime, therefore they were excluded from further analysis.

### Blood Glucose Levels

**Figure 3:**
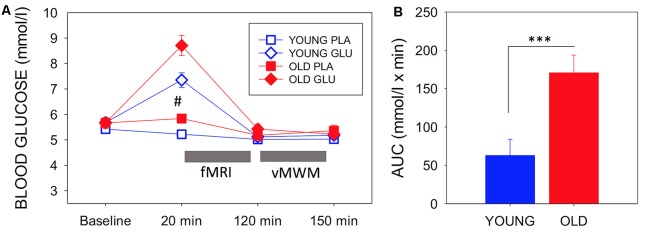
**(A)** Mean (with SEM) blood glucose levels at baseline, pre-magnetic resonance imaging (MRI) (20 min post-dose), post-MRI (120 min post-dose), and end of testing (150 min post-dose) for each group and each visit. Circles depict younger adults, while squares depict older adults. Filled symbols represent measures taken on glucose visit, while open symbols represent measures at the placebo visit. ^#^Indicates a significant difference between drink condition and significant difference between ages in both drink conditions (*p*-values see text). Timing of fMRI and virtual Morris Water maze (vMWM) are indicated in relation to glucose measurements. **(B)** Blood glucose incremental area under the curve as a measure of glucoregulatory efficiency, bars depict mean (with SEM). Older adults had significantly higher incremental area underthe curve (iAUC) than younger adults (*T*_(1,28)_ = −3.403, *P* = 0.002), indicating poorer glucose regulation. ***P < 0.005.

Blood glucose levels throughout both testing sessions for each group are depicted in Figure 3A, which also presents the timing of the experimental measures. There were no between-group differences in baseline blood glucose levels between the younger and older group either at glucose (*T*_(1,28)_ = 0.59, *P* = 0.954) nor placebo visit (*T*_(1,28)_ = −0.878, *P* = 0.387).

A 2 (Age: young/old) × 2 (Treatment: Glucose/Placebo) × 4 (Timepoint: 0, 20, 120, 150 min) ANOVA was conducted. The rmANOVA analysis of glucose levels omitted data from one young participant who had a missing post-dose blood glucose assessment at the placebo visit.

There was a significant main effect of Treatment (*F*_(1,27)_ = 25.18 *P* < 0.001, *η*^2^ = 0.485), a main effect of Time point (*F*_(1.567,27)_ = 59.43, *P* < 0.001, *η*^2^ = 0.688, Greenhouse-Geisser) and a significant Treatment × Timepoint interaction (*F*_(1.843,27)_ = 19.43, *P* < 0.001, *η*^2^ = 0.644, Greenhouse–Geisser). There was also a main effect of Age (*F*_(1,27)_ = 9.076, *P* = 0.006, *η*^2^ = 0.252). The older group showed a greater increase of blood glucose levels in response to glucose ingestion than the younger group (*T*_(1,28)_ = −2.99, *P* = 0.006).

There was also a significant difference in blood glucose levels 20 min post-ingestion at the placebo visit (*T*_(1,27)_ = − 3.72, *P* = 0.001). Glucose levels decreased in the younger group.

Using blood glucose iAUC at the glucose visit as a measure of glucoregulatory efficiency, older participants had significantly higher iAUC than younger participants (*T*_(1,28)_ = −3.403, *P* = 0.002), indicative of poorer glucose regulation in the older sample (Figure 3B).

### Resting-State

**Figure 4:**
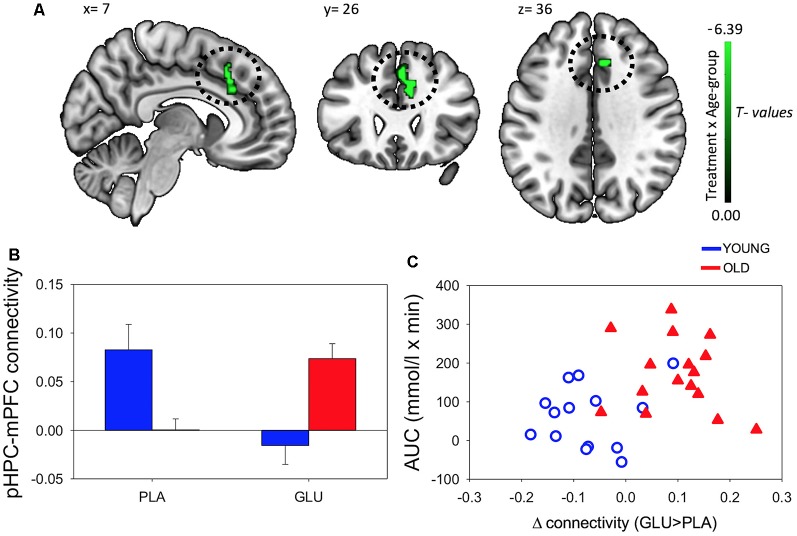
**(A)** Resting-state functional connectivity (rsFC) brain map for left posterior hippocampus (pHPC) showing: Treatment × Age-group interactions exhibited from left pHPC to cluster in medial frontal cortex (mPFC; encompassing anterior cingulate, paracingulate gyrus and superior frontal gyrus; fisher z-transformed correlation values). **(B)** Extracted connectivity strength from pHPC to mPFC for each group per session (error bars reflect SEM). **(C)** Scatterplot of correlation between change in rsFC of pHPC and mPFC and glucose regulation as measured by iAUC (*r* = 0.39, *P* = 0.04).

A significant Treatment × Age-group interaction in rsFC was observed between left pHPC and a cluster within in the medial frontal cortex (mPFC) encompassing areas in anterior cingulate, paracingulate gyrus and superior frontal gyrus [Brodmann Area (BA) 32 and 8; MNI peak (+08 +26 +30; Table 2 and Figure 4A)]. There were no main effects of condition or group.

**Table 2:** Whole-brain voxel-wise rmANOVA of resting-state functional connectivity (rsFC) with left posterior hippocampus.

Cluster *p*	Cluster size	Peak	MNI co-ord (peak)			Peak region
(FWE-corr)	*k* voxels	*t*	*x*	*y*	*z*	
0.044	96	−6.39	8	26	30	Paracingulate gyrus R

*Treatment × age-group interaction (young > older; Glucose > Placebo) for left posterior hippocampus (pHPC) seed (two-sided contrast; 5,000 permutations, FWE-corrected) to cluster in mPFC (encompassing anterior cingulate, paracingulate gyrus and superior frontal gyrus). The table displays cluster size p-value (FWE-corr), cluster size (k), Peak t-value, Montreal Neurological Imaging (x, y, z) peak coordinates, peak region (Harvard-Oxford Atlas), R = right hemisphere*.

*Post hoc* *t*-tests revealed that glucose (compared to placebo) significantly increased left pHPC-mPFC connectivity in older participants, *T*_(1,15)_ = 5.13, *P* < 0.001), whereas the reverse was observed in young participants, *T*_(1,13)_ = −3.6, *P* = 0.003 (Figure 2B).

This analysis was repeated using individual pHPC volume as a covariate. The cluster in the same area remained significant although smaller in size [MNI peak (+04 +24 +44); Voxels_(k)_ = 58]; see section 3.2 in Supplementary Material, Supplementary Table S3.2). No significant differences were observed from any of the other seed ROIs (right pHPC, or left and right aHPC).

Further *post hoc* tests showed that younger participants exhibited higher rsFC between left pHPC and mPFC relative to older participants under placebo conditions, *T*_(1,28)_ = 3.54, *P* = 0.001; and that older participants exhibited significantly greater left pHPC-mPFC connectivity relative to the younger group after glucose ingestion alone, *T*_(1,28)_ = −2.75, *P* = 0.01 (Figure 4B).

To relate the present finding to individual glucose regulation, *post hoc* Pearson correlation using iAUC at the glucose treatment visit and rsFC connectivity were performed. Change in pHPC-mPFC rsFC was correlated with individual glucose regulation across the whole sample (*r* = 0.39, *P* = 0.04; Figure 4C).

### Task Performance

**Figure 5:**
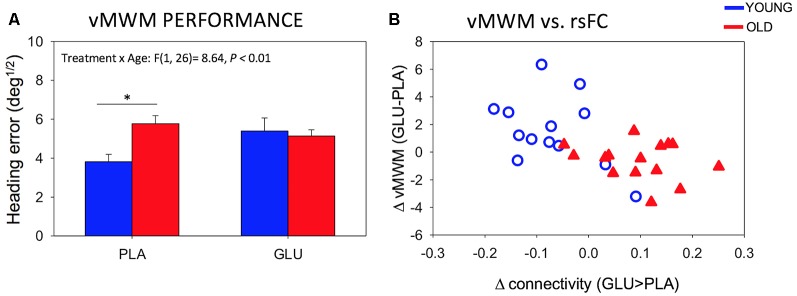
**(A)** Behavioral performance on the vMWM task for younger and older subjects following placebo and glucose drink. Bars depict mean Heading error (deg^1/2^; with SEM). Smaller values reflect better performance *P < 0.05. **(B)** Scatterplot of change in rsFC of left posterior hippocampus to mPFC and change in performance on the spatial navigation task (heading error; *r* = −0.56, *P* = 0.002).

Data from two additional participants (one young, one old) was omitted from the analysis of the vMWM due to missing data. There was a significant Treatment × Age-group interaction for performance on the vMWM task (*F*_(1,26)_ = 8.64, *P* = 0.007) as depicted in Figure 5A. *Post hoc* pairwise comparisons revealed that while the older group showed significantly worse performance compared to the younger group under placebo (*T*_(1,26)_ = −3.42, *P* = 0.02), there was no group difference after glucose administration (*T*_(1,26)_ = 0.35, *P* = 0.73). No Treatment by age-group interactions were found in the Serial Sevens (correct; *F*_(1,28)_ = 1.38, *P* = 0.25).

We further probed the relationship between significant changes in left pHPC-mPFC rsFC and change in performance on the spatial navigation task. Change in rsFC magnitude between left pHPC and mPFC was correlated with changes in spatial memory performance (*r* = −0.56, *P* = 0.002). The negative correlation reflects greater connectivity after glucose to be associated with smaller heading errors (averaged across the two probe trials) after glucose ingestion. Specifically, in older participants, glucose increased functional connectivity between the left posterior hippocampus and mPFC and that the magnitude of this functional connectivity change correlated with the change in performance (Figure 5B).

## Discussion

The aim of the present study was to investigate age-related differences in changes in rsFC in response to a glucose load from the anterior and posterior segments of the hippocampus, comparing a group of younger and a group of older adults. Further, we wanted to investigate if these changes are linked to individual glucose regulation and how they relate to memory performance on two different memory tasks.

After the ingestion of a 25 g glucose drink, we observed changes in functional connectivity of the pHPC to a cluster in the mPFC. In older participants, glucose increased rsFC between pHPC and mPFC. Furthermore, the change in connectivity was related to glucoregulatory ability. Participants with poorer glucose regulation, as indicated by greater circulating glucose following administration, benefited most from the glucose load. In younger participants we observed the opposite pattern of connectivity change. Moreover, the change in the magnitude of rsFC was correlated with gains in performance on a spatial memory task.

Here, we demonstrate age-dependent, acute modulation of pHPC connectivity, suggesting that connectivity of the posterior segment of the hippocampus is differentially susceptible to an acute glucose load in older individuals.

This study is the first to compare the effects of glucose on rsFC and cognition in both younger and older individuals. There is a general consensus that glucose enhancement is more effective in older compared to younger adults (Macpherson et al., 2015). This effect has been partially attributed to an age-related decline in glucose regulation. Our findings indicate that pHPC-mPFC connectivity increases were more marked as glucoregulation worsened in older participants (see Figure 4C). Our results suggest that these connectivity changes may contribute to previously reported demonstrations that glucose more readily attenuates age-related cognitive performance decrements in elderly adults with impaired glucose regulation (Kaplan et al., 2000; Messier et al., 2003).

The hippocampus has been hypothesized as a key structure in the glucose facilitation effect (Smith et al., 2011). Our results show the potential importance of considering subdivisions of the hippocampus along its anterior-posterior axis in neuropharmacological studies.

The hippocampus and surrounding temporal lobes have long been recognized to be an important node for the processing of spatial information (for a review, see Howard et al., 2005). The results of the present study support the role of the posterior segment of the hippocampus in spatial memory performance. This relationship has recently been demonstrated in a rsfMRI study by Persson et al. (2018), who predicted spatial memory performance from pHPC, but not aHPC, rsFC. Our results are also consistent with reports of positive associations between posterior hippocampal activation and virtual navigation performance (Maguire et al., 1998; Hartley et al., 2003; Cornwell et al., 2008).

Growing evidence points to the importance of the mPFC, including the anterior cingulate cortex (ACC), in spatial memory. Interactions between mPFC and hippocampus have been proposed to be critical to successful encoding and retrieval of spatial information (Wirt and Hyman, 2017). The present results show that increases in pHPC-mPFC rsFC are beneficial to navigation task performance, thus contributing to the growing amount of literature emphasizing the importance of information sharing between these two areas in spatial memory.

The post-glucose performance of the older participants was similar to that of the younger group. Conversely, in the younger group performance decreased slightly. In young adults, the glucose facilitation effect is most readily observed under increased task difficulty (Kennedy and Scholey, 2000; Scholey et al., 2001) and divided attention (Sünram-Lea et al., 2002; Scholey et al., 2009). It is thus possible that the task demands in this study were not high enough to elicit an effect in the younger group. It is also the case that there are a number of studies where glucose modulation did not affect behavioral task performance (Knott et al., 2001; Riby, 2004), and there are isolated reports of decreased task performance after glucose ingestion (Scholey et al., 2015). This may be a manifestation of complex interactions between glucose levels and task demands which make the effect more fragile in younger adults.

The reason for the difference between blood glucose levels 20 min post-ingestion of the placebo drink are unknown, but they may reflect better glucoregulation as reflected intact insulin signaling after (false) nutritional load in the young group. Anticipatory hormonal response to flavoring (without glucose) has been observed before (Scholey and Kennedy, 2004).

There were some limitations to this study. The sample sizes were relatively small, and future studies with larger sample sizes are needed in order to investigate the glucose facilitation effect especially with regard to mediating variables such as gender. There is research suggesting that gender might be a contributing factor in the atrophy of the hippocampus (Pruessner et al., 2001) as well as a factor in the glucose facilitation effect (Craft et al., 1994). Even though gender-matched cohorts were used in the current study, larger sample sizes are necessary to increase the power to detect potential gender differences. Future studies investigating the effects of gender and other mediating variables are encouraged.

The consumption of glucose results not only in increases in glucose levels but also increases in systemic insulin and gut peptides, such as glucagon-like peptide-1 (GLP-1), cholecystokinin (CCK) and peptide tyrosine-tyrosine (PYY; Zanchi et al., 2017). In the current investigation we did collect endocrine data, which limits the interpretation of results. As the timing of cognitive task was 2 h post glucose ingestion it could be argued that the effect was driven by changes in insulin levels. Cognitive enhancing properties of insulin have been revealed in previous investigations (Benedict et al., 2004; Krug et al., 2010). A recommendation for future studies is, therefore, to include measurements of insulin and gut peptides to examine the cognitive enhancing mechanism of glucose ingestion in more detail.

We used a hypothesis-driven seed-based analysis to investigate the role of the anterior and posterior segments of the hippocampus in the glucose facilitation effect. This kind of analysis is influenced by the seed coordinates chosen. The seed coordinates for the anterior and posterior hippocampus in the present study were determined based on other investigations (Wagner et al., 2016) to facilitate comparisons between studies. However other researchers have used different seed coordinates for anterior and posterior hippocampus (e.g., Damoiseaux et al., 2016) which may affect the results.

## Conclusion

This is the first study investigating the functional connectivity of aHPC and pHPC after glucose load in young and elderly adults.

The results of the present study indicate the possibility that the pHPC is especially sensitive to pharmacological interventions, as we have shown that a simple glucose load modulates its connectivity and enhances cognitive performance in older adults.

The results also suggest that glucose modulated functional connectivity and cognitive performance more readily in older adults with impaired glucose regulatory ability. We further demonstrated the functional relevance of the changes in functional connectivity by relating gains in performance on a spatial memory task to increase in pHPC-mPFC connectivity.

## Data Availability Statement

The data that support the findings of this study are available on reasonable request from the corresponding author (RP). The SPMs of all reported contrasts (and their unthresholded versions) are available at: https://neurovault.org/collections/TRYHPFXR/.

## Ethics Statement

The studies involving human participants were reviewed and approved by Swinburne University Ethics committee. The patients/participants provided their written informed consent to participate in this study.

## Author Contributions

AS and DW designed the experiment and supervised all aspects of the study. BC and RP contributed to the design of the study. RP collected and analyzed the data. DW and BC contributed to data analysis. RP prepared the manuscript. DW, AS and BC helped to draft and revise the manuscript. All authors have read and approved the manuscript.

## Conflict of Interest

DW has received research funding and/or consultancy/speaker honoraria from Abbott Nutrition, Arla Foods, Bayer Healthcare, and Neurobrands. AS has received research funding and/or consultancy/travel/speaker fees from Abbott Nutrition, Australian Research Council, Arla Foods, Australian Wine Research Institute, Barilla, Bayer, Biotechnology and Biological Sciences Research Council, Blackmores, Cognis, Cyvex, Dairy Health Innovation Consortium, Danone, DuPont, European Commission Framework 5 Research and Innovation initiative, GlaxoSmithKline, Ginsana, Kemin Foods, Martek, Masterfoods, National Health and Medical Research Council, Naturex, Nestlé, Neurobrands, Novartis, Nutricia-Danone, Red Bull, Sanofi, Sen-Jam Pharmaceuticals, Verdure Sciences, Unilever, Wrigley Science Institute. The remaining authors declare that the research was conducted in the absence of any commercial or financial relationships that could be construed as a potential conflict of interest.

## Footnotes

^1^http://www.nitrc.org/projects/conn

^2^http://marsbar.sourceforget.net/
